# Environmental drivers of tropical forest snake phenology: Insights from citizen science

**DOI:** 10.1002/ece3.10305

**Published:** 2023-07-23

**Authors:** Letízia M. G. Jesus, Jhonny J. M. Guedes, Mario R. Moura, Renato N. Feio, Henrique C. Costa

**Affiliations:** ^1^ Museu de Zoologia João Moojen, Instituto de Ciências Biológicas e da Saúde Universidade Federal de Viçosa Viçosa Brazil; ^2^ Programa de Pós‐Graduação em Biodiversidade e Conservação da Natureza, Instituto de Ciências Biológicas Universidade Federal de Juiz de Fora Juiz de Fora Brazil; ^3^ Programa de Pós‐Graduação em Ecologia e Evolução, Departamento de Ecologia, Campus Samambaia Universidade Federal de Goiás Goiânia Brazil; ^4^ Departamento de Biologia Animal Universidade Estadual de Campinas Campinas Brazil; ^5^ Departamento de Ciências Biológicas Universidade Federal da Paraíba Areia Brazil; ^6^ Departamento de Zoologia, Instituto de Ciências Biológicas Universidade Federal de Juiz de Fora Juiz de Fora Brazil

**Keywords:** Atlantic forest, circular analyses, citizen science, climate change, local collectors, phenology, venomous snakes

## Abstract

Museum specimens and citizen science initiatives are valuable sources of information on how anthropogenic activities affect biodiversity and how species respond to rapid global change. Although tropical regions harbor most of the planet's biodiversity, investigations on species' phenological changes are heavily biased toward temperate regions. Such unevenness in phenological research is also taxonomically biased, with reptiles being the least studied group among tetrapod species regarding animal phenology. Herein, we used long‐term time‐series data to investigate environmentally driven changes in the activity pattern of tropical forest snakes. We gathered natural history collection and citizen science data for 25 snake species (five venomous and 20 non‐venomous) from an Atlantic Forest region in southeastern Brazil. Using circular mixed‐effects models, we investigate whether snake activity patterns followed the variation in environmental variables over a decade. Our results show that the activity pattern of Atlantic Forest snakes was seasonal and largely driven by average temperature and relative humidity. Since snakes are ectothermic animals, they are particularly sensitive to temperature variations, especially at small scales. Moreover, relative humidity can affect snake's seasonal activities through physiological constraints and/or prey availability. Most specimens were registered during the rainy season, with highly venomous snakes (lanceheads and coral snakes) emerging as the most abundant taxa. We highlight the importance of citizen science and natural history collections in better understanding biodiversity. Furthermore, our data obtained from local collectors underscore the need for environmental education programs and collaboration between researchers and local decision‐makers to raise awareness and reduce conflicts between people and snakes in the region.

## Graphical abstract

We used long‐term time‐series data to investigate the environmentally driven changes in the activity patterns of 25 snake species in the Atlantic Forest region of southeastern Brazil. Overall, we found snake activity patterns were largely driven by temperature and humidity, with venomous and non‐venomous species responding differently to climate. Our findings can have implications for local health policy, conservation, and management efforts.

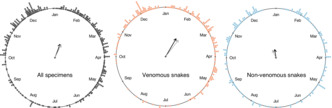

## 1 INTRODUCTION

Specimens deposited in natural history collections are an important source of biodiversity data, representing temporal and spatial snapshots of, for example, species occurrences, rarity, and distribution (da Silva et al., 2018; Davis et al., 2015; Marinoni & Peixoto, 2010). However, only a small fraction of global biodiversity is covered by systematic sampling and fieldwork experiments, usually resulting in high spatial clustering of available occurrence records (Meyer et al., 2015). In this sense, citizen science initiatives have become an extremely valuable source for biodiversity information (Chandler et al., 2017; Theobald et al., 2015) because data derived from both preserved specimens and citizen science may reduce existing biases, being crucial to understand, for example, how species respond to rapid global change (Meineke et al., 2019; Pocock et al., 2018). When accumulated across large spatial or temporal scales, the information available through preserved specimens and citizen science helps to inform research on how species shift their phenology, morphology, and geography across years or even centuries (Garbino et al., 2022; Lamichhaney et al., 2019; Magioli et al., 2020; Moura et al., 2022). Surprisingly, these data sources are heavily underused in global change research (Chandler et al., 2017; Peter et al., 2019; Sanders et al., 2023).

Although digitized information on preserved specimens allows investigations on many aspects of species geography (Tingley & Beissinger, 2009), some issues may arise due to challenges in data quality (Vollmar et al., 2010). For instance, major online databases on preserved specimens, such as the Global Biodiversity Information Facility (GBIF; https://www.gbif.org/) and the SpeciesLink Network (https://specieslink.net/), inform only the month and year of collection, either due to a lack of correct digitalization of the data or by real absence of georeferenced collection information. While these records are useful to study species' biogeography (considering that coordinates or locality data are available), they are unsuitable for phenological research. The lack of exact collection dates can hinder research on climate change‐driven shifts in phenology, since most changes observed in species phenology comprehend the interval of a couple of days per decade (Cohen et al., 2018; Parmesan & Yohe, 2003). To worsen this scenario, the current knowledge on species phenology is largely biased toward temperate regions and taxa that are charismatic and/or easy to observe, such as birds and butterflies (e.g., Carr et al., 2021; Faltýnek Fric et al., 2020; Lafferty, 2001). For instance, among the 394 time series compiled for a recent global synthesis on species phenology (Cohen et al., 2018), none was located in South America or Africa, and while 92% of those available time series concerned tetrapod taxa, only 0.5% referred to reptiles (Cohen et al., 2018), by far the least represented tetrapod group.

Herein, we use long‐term time‐series data derived from a collaboration between a natural history museum and a citizen science initiative to assess how phenological activities in tropical forest snakes respond to different environmental variables. Specifically, we investigate whether activity patterns of venomous and non‐venomous snakes follow the seasonal climate of the Brazilian Atlantic Forest hotspot (Zachos & Habel, 2011) over a decade, and assess which abiotic factors (if any) affect species phenology. Phenological investigations on Atlantic Forest snakes are scarce and available studies have often focused on coastal localities of dense evergreen rainforests, with high, but less seasonal rainfall compared with inland areas (Barbo et al., 2011; Marques et al., 2001, 2006; Parpinelli & Marques, 2008). Most of these studies lack long‐term time‐series data and only investigate daily and annual seasonality patterns, disregarding potential changes in phenology over the years. Since phenological activity in tropical species are mostly driven by rainfall (Cohen et al., 2018; Marques et al., 2001; Winter et al., 2016), it is possible that species living in semidecidual rainforests—subject to more seasonal climates—have experienced phenological shifts due to climate change in the region. Snakes are particularly sensitive to climate change due to their small dispersion capabilities and their ectothermic physiology (Cohen et al., 2018; Sahlean et al., 2014; Segura et al., 2007). In temperate areas, for instance, temperature rise and the resulting reduction in humidity and rainfall have been found to directly affect locomotion, thermoregulation, foraging, predation, dial, and seasonal activity in snakes (George et al., 2015; Rugiero et al., 2013; Sahlean et al., 2014). Lastly, considering that periods of high snake activity patterns are associated with snakebite incidence (Schneider et al., 2021; World Health Organization, 2018), understanding the temporal dynamics of snake phenology can help to improve health planning policies and minimize snake envenomation (Melo Araújo et al., 2022; Minghui et al., 2019; The Lancet, 2017).

## 2 METHODS

### 2.1 Study area

**FIGURE 1:**
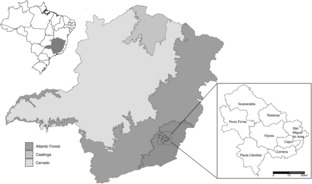
Map of the study area. Viçosa and its seven neighboring municipalities are inserted in the state of Minas Gerais, Brazil.

Our study took place in the municipality of Viçosa (20°45′S, 42°52′W, 648 m) and its neighboring cities (Figure 1), state of Minas Gerais, southeastern Brazil. The region is characterized by semidecidual seasonal rainforest vegetation (Coelho et al., 2005), but its original coverage has been significantly reduced by land use change for farming and livestock, as well as urban expansion (Coelho et al., 2005; Costa et al., 2010; Ribon et al., 2003). The climate in the study area is humid subtropical with rainy summers (October to April) and dry winters (May to September); the mean annual temperature is 20.1°C, and the mean annual rainfall is 1289.0 mm (Anon, 2022; Martins et al., 2018).

### 2.2 Data collection

**T**he reptile collection of the Museu de Zoologia João Moojen, at Universidade Federal de Viçosa (MZUFV), currently holds 2780 preserved specimens of snakes. Many snake specimens at the MZUFV were obtained through efforts from local collectors (i.e., firefighters, environmental police, and local people) over the years, although not all the donated specimens have been deposited in the MZUFV collection. Some snakes were released back into the wild, but not without registering information on species name, location, time, and date of capture. This citizen science initiative, in which local collectors refrained from killing snakes and instead brought them to the museum, has provided taxonomically verified records of snake activity since January 2009. We used all information gathered up to December 2018 in this study, representing a 10‐year time‐series data on snake reception dates as a proxy for snake seasonal activity.

We acknowledge that using such data as a proxy for activity patterns may come with some caveats. Firstly, most specimens are brought alive to the museum, meaning that snakes that are killed every year by local people in the region rarely reach the scientific collection. Secondly, it is possible that the peaks of snake activity observed in our study could reflect human behavior, such as increased outdoor activities during the coffee harvest season in the region. However, we stress that most specimens received at the MZUFV by citizen scientists were mainly from urban and peri‐urban environments, not rural areas where coffee is harvested (Costa et al., 2010). Most specimens found by coffee farmers are likely killed and disposed of in situ and not brought to the museum; Therefore, the impact of coffee harvest on the observed patterns is unlikely. Lastly, in contrast to temperate regions where snow and low temperatures can restrict human activity during winter, our study area is located in a tropical environment where such constraints are absent. In summary, while there are some limitations associated with our data, the observed patterns are likely to reflect snake activity in the region and the associated probabilities of being found and donated to the sole museum in the area.

**FIGURE 2:**
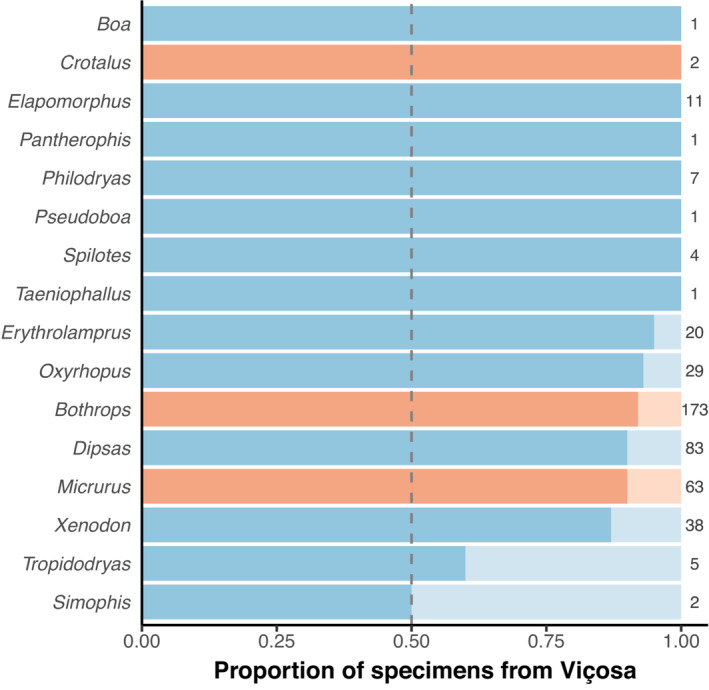
Proportion of specimens registered in the Viçosa municipality or neighbor cities across snake genera. Bluish colors indicate non‐venomous (non‐ or rear‐fanged) snakes, whereas reddish colors denote venomous (front‐fanged) species. Darker hues show the proportion of specimens from the municipality of Viçosa. Numbers at the end of the bars show the total number of specimens per species. Genera with less than three recorded specimens were not included in the circular mixed‐effects models (see Section 2).

**FIGURE 3:**
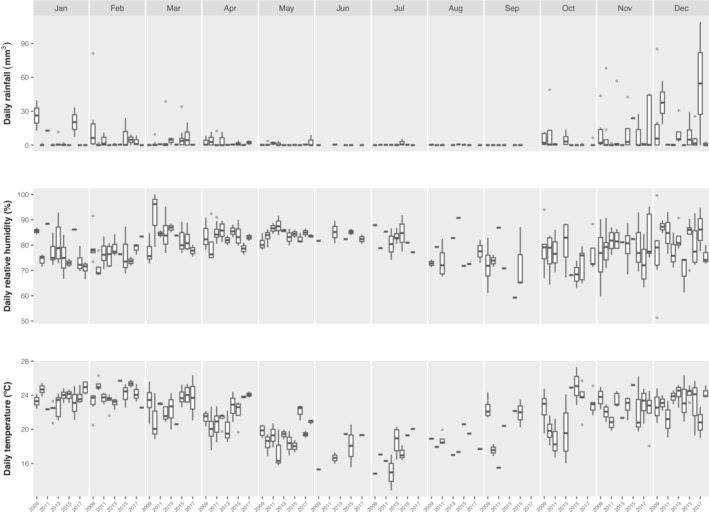
Temporal variation among environmental variables over a decade. Data were extracted from Instituto Nacional de Meteorologia (https://portal.inmet.gov.br/dadoshistoricos) and are based on the weather station from Viçosa. Daily data were grouped by month and year, and filtered to include only days on which snakes were brought by the local people to the museum of zoology.

Since most snakes received at the MZUFV during this period are from Viçosa municipality (c. 80%; Figure 2; see the raw data in the Appendix S1 for the municipality of collection of each specimen), we collected the city's weather data from 2009 to 2018 (Anon, 2022) for subsequent analysis (see below). We computed the daily average temperature by averaging the daily average maximum and minimum temperatures and obtained data on daily average relative humidity and daily total precipitation (Figure 3). Since people are likely less active during rainy days, it could decrease the probability of encounters with snakes and blur the potential impact of precipitation (based on daily values) on snake activity. To check this potential bias, we performed a sensitivity analysis by rerunning our model (see model specifications below) using the sum of precipitation values for the week prior to the snake capture date.

### 2.3 Statistical analysis

We used circular analyses (Pewsey et al., 2013; Zar, 1984) to investigate snake's seasonal activity. We converted collection dates (in Julian days) by dividing them by 365 (or 366 for data collected in the leap‐years of 2012 and 2016) and then multiplying by 360 (representing degrees) (Pewsey et al., 2013). We used the angular frequencies to analyze whether snake reception data were concentrated in some period (indicating seasonality) and to investigate which environmental factors may influence observed patterns. We provide descriptive circular statistics with (1) the circular mean and median, which shows the period in which more snakes were active, (2) the mean resultant length, which shows if data are more dispersed (*r* = 0) or concentrated (*r* = 1) in any direction, (3) the circular variance, and (4) the circular standard deviation (Table 1). We performed (i) a Watson's test to check if the overall circular data fits the von Mises (normal) distribution, and (ii) a Rayleigh test of uniformity (or a Watson's non‐parametric test in case the data are not normal) to test for directionality.

**TABLE 1:** Descriptive circular statistics for the snake reception data with mean and median direction, mean resultant length (MRL), circular variance (*V*_m_), and circular standard deviation (*v*) for all specimens combined, as well as for highly venomous (of medical importance) and non‐ or slightly venomous snakes separately. Note that degrees can be roughly interpreted as current days so that a mean resultant length of 20° roughly represents January 20th, while 351° would fall in late December.

Sample set	*N*	Mean	Median	MRL	*V*_m_	*v*
Viçosa‐only						
All specimens	402	20.55°	25.64°	0.29	0.71	1.57°
Venomous	218	30.1°	37.48°	0.42	0.58	1.32°
Non‐venomous	184	351.45°	335.34°	0.17	0.83	1.88°
Viçosa and vicinities						
All specimens	441	21.75°	25.64°	0.3	0.7	1.56°
Venomous	238	32.73°	38.41°	0.42	0.58	1.32°
Non‐venomous	203	352.07°	335.34°	0.19	0.81	1.83°

We built circular mixed‐effects models (Cremers et al., 2018) to test the influence of environmental variables (daily average temperature, daily average relative humidity, and daily total precipitation—as well as summed precipitation values over the previous week in a sensitivity analysis) upon day of snake activity (in radians) with genus identity as a random effect. Prior to the analysis, we applied a cube root transformation to precipitation to reduce skewness and kurtosis. We checked for multicollinearity among predictors using the variation inflation factors (VIF; Mansfield & Helms, 1982), where strong multicollinearity is usually attributed to VIF values >10 and indicates that variables should not be included in the analysis (Kutner et al., 2005). Since none of our continuous variables reached VIF > 2, we kept them all (Table S1). We also added a categorical variable informing whether species are of medical importance (highly venomous pitvipers and coral snakes) or not (non‐venomous [not‐fanged] / mildly venomous [rear‐fanged] snakes) to test for potential differences in activity between them given the medical implications of venomous snakes in public health. We note that most venomous snakes in the region are sit‐and‐wait predators, while most non‐venomous snakes actively search for their prey, which could also influence the observed patterns. Lastly, we built a new model to test the potential and isolated effect of collection year upon snakes' reception dates to assess whether snake activity has shifted throughout a decade in the region. For all models, genera with less than three sampled specimens were removed from the dataset to reduce instability in model estimates (Harrison et al., 2018). Taxonomy follows the Brazilian List of Reptiles (Costa et al., 2022), but in some cases, the specimen received was identified at the reception record only to genus level (see raw data in the Appendix S1).

The models were fitted using a Bayesian approach and Markov Chain Monte Carlo (MCMC) sampler to estimate model parameters. MCMC methods are iterative; thus, we ran 10,000 iterations with a burn‐in period of 100 iterations and a lag of three to prevent possible autocorrelation between parameter estimates (Cremers et al., 2018). Model convergence was checked through traceplots. To assess model fit, we built intercept‐only models that were used as a baseline for comparison with the “full” models (i.e., containing predictor variables). We show model fit values (see Table S2) using four different model fit criteria: two versions of the deviance information criterion (DIC) and two versions of the Watanabe‐AIC. We also checked whether our predictors explained a substantial part of the random effect variances by comparing the posterior estimates of the circular random intercepts for the intercept‐only models and the full models (Cremers & Klugkist, 2018). The effect sizes of z‐transformed continuous predictors were represented by the “slope at the mean” (SAM) circular coefficients (Cremers et al., 2018), which inform how angular dates of snake activity are affected by the respective standardized predictor. Because most specimens in our dataset and the weather station monitoring data were both obtained from the Viçosa municipality, we performed the analysis using only specimens collected there. We also re‐run the analysis using all specimens from Viçosa and its neighboring cities (see Appendix S1), while looking for potential differences in model outputs. We conducted all analyses in the software R version 4.0.2 (R Core Team, 2020) using the packages *circular* 0.4.95 (Agostinelli & Lund, 2017) and *bpnreg* 2.0.2 (Cremers, 2021).

## 3 RESULTS

Over a decade, local collectors brought 479 snakes from 23 municipalities to the MZUFV (mean = 47.9 snakes/year, range: 28–69 snakes). Snakes received belong to 17 genera and 25 species. When we considered only specimens from Viçosa and its seven neighboring cities, the total number of snakes decreased to 441 (mean = 44.1 snakes/year, range = 27–63 snakes), with most snakes belonging to the families Dipsadidae (false coral snakes, ground‐snakes, racers, snail‐eaters, and others; *n* = 195; 44.2%) and Viperidae (lanceheads and rattlesnakes; *n* = 175; 39.7%), followed by Elapidae (coral snakes; *n* = 63; 14.3%), Colubridae (rat‐snakes and sipos; *n* = 7; 1.6%), and Boidae (boas; *n* = 1; 0.2%). The most common snakes in Viçosa and vicinities were the jararaca *Bothrops jararaca* (*n* = 125, 28.3%), the southern coral snake *Micrurus frontalis* (*n* = 55; 12.5%), the Brazilian slug‐eating snake *Dipsas mikanii* (*n* = 52, 11.8%), and the jararacuçu *Bothrops jararacussu* (*n* = 44, 10%), representing nearly two‐thirds of all recorded snakes. We found two non‐native species in Viçosa, *Pantherophis guttatus* (*n* = 1), and *Boa constrictor* (*n* = 1, which, despite being a native species in Brazil is not considered native to Viçosa). In total, 54% (*n* = 238) of the snakes' specimens received are medically important, that is, highly venomous pitvipers and coral snakes.

**FIGURE 4:**
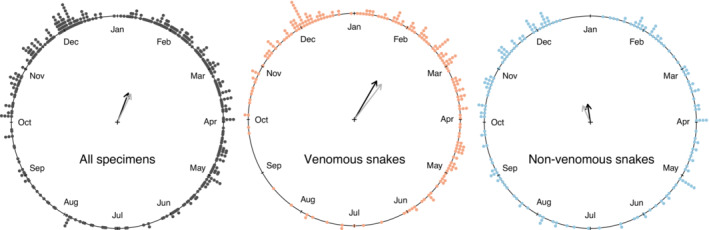
Snake reception dates based on local collectors' efforts in the interior Atlantic Forest of southeastern Brazil. Circular plots show the distribution of all snakes (left) as well as venomous (center) and mildly/non‐venomous snakes (right) received at the MZUFV collection throughout the calendar year over a decade. The arrows show the median (gray) and mean (black) direction, and the mean resultant length (a concentration metric), where shorter arrows indicate more dispersed snake reception dates. Data is from specimens collected in Viçosa‐only.

Most specimens were captured and brought to the museum by local collectors from October to April (Figure 4 and Figure S1), highlighting a seasonal activity pattern in the region (Watson's uniformity test: statistics = 1.88, *p* < .01). The activity of highly venomous snakes did not differ significantly from that of non‐venomous / mildly venomous snakes (Figure 4 and Figure S1, Table 1), with great overlap in their capture dates (non‐significant differences in posterior modes (PM) of circular coefficients = −0.29° [lower bound = −1.11; upper bound = 0.66°]).

We found that snake activity patterns in Viçosa were positively associated with daily average temperature (PM = 2.03° [0.92 to 8.39]) and daily relative humidity (PM = 1.76° [0.93 to 3.09]; Table 2), that is, the lower and upper bounds for the circular coefficients did not embrace zero. Conversely, there was no significant effect of daily precipitation (PM = −1.45° [−11.1 to 7.16]) nor of the summed values of precipitation in the week prior to the date of capture on snake activity (Table S3). When running the model with specimens from Viçosa and surrounding municipalities, model outputs were virtually the same (Table 2). The environmental variables explored in these models greatly improved model fit (Table S2) and helped to explain most of the random intercept variance, reducing it by 327 and 10 times, respectively, compared with baseline intercept‐only models. We found no evidence of any significant changes in average snake reception dates throughout the study period in the region (PM = 0.001° [−0.47 to 0.41]).

**TABLE 2:** Results from the circular mixed‐effect models.

Sample set	Predictors	Mode	SD	LB	UB
Viçosa‐only	Precip.z	−1.45 (−83.1)	118.6	−11.10	7.16
	AvgTemp.z	2.03 (116.7)	19.4	**0.92**	**8.38**
	RelHumid.z	1.77 (101.3)	0.6	**0.94**	**3.10**
Viçosa and vicinities	Precip.z	−1.36 (−78.2)	21.9	−11.70	6.82
	AvgTemp.z	1.94 (111.1)	52.6	**0.98**	**6.88**
	RelHumid.z	1.69 (96.8)	0.6	**0.97**	**2.95**

*Note*: Posterior modes in radians (and degrees) and 95% highest posterior density (HPD) interval, with lower (LB) and upper bounds (UB) for the circular regression SAM coefficients for the continuous variables of the snake reception data. Bold values indicate that an HPD interval does not contain 0. Predictors were z‐transformed to make them comparable.

## 4 DISCUSSION

By making use of data from specimens captured by citizen scientists, we show that snakes from the interior Atlantic Forest of southeastern Brazil have a seasonal activity pattern, being more active in the rainy season. Daily temperature and relative humidity positively influence snake activity in the region, while daily rainfall was unimportant. We also found that among the most common species recorded over a decade by local collectors, most are highly venomous snakes that may benefit (i.e., increase their geographic range or activity period) from human disturbances in the environment. Citizen scientists also recorded non‐native species in the region, which were possibly kept as pets for some time and then released in the wild by their owners. Lastly, over the 10‐year time series, we found no pronounced phenological shifts in snake activity over time.

The most important predictor of snake activity in the interior Atlantic Forest was daily average temperature. Snakes are ectothermic animals whose physiology and behavior as well as phenology are heavily affected by environmental temperature (Buckley et al., 2012; Deutsch et al., 2008; Rugiero et al., 2013). Such temperature dependence is more acute in temperate regions, where harsher and more extreme environmental conditions increase, for example, energy requirements for foraging and reproduction (Lillywhite, 1987; Moreno‐Rueda et al., 2009). We show that daily temperatures also play a major role on phenology patterns of snake communities in the interior Atlantic Forest, even though the temperature is more constant and less variable throughout the year (Figure 3). The influence of temperature in seasonal activity of snakes varies along the Atlantic Forest, probably due to an interaction with biotic (prey availability and predator activity) and other abiotic variables (e.g., latitude and elevation). In coastal Atlantic Forest, most species are less active in periods of less rainfall, which coincides with lower temperatures in forest microhabitats (Marques et al., 2001; Pontes et al., 2009). But in the southern Atlantic Forest, where variation in temperature along the year is more pronounced, snakes are usually more active in warmer months (Rocha et al., 2014; Zanella & Cechin, 2009).

Snake phenology was positively influenced by daily relative humidity in the study region. Relative humidity affects snake seasonal activities, particularly in assemblages located in areas with a well‐marked dry season (Henderson et al., 1978; Martins & Oliveira, 1998; Strüssmann & Sazima, 1993), mostly through physiological constraints related to dehydration, as well as prey availability (Abom et al., 2012; Kearney et al., 2013). Due to synoptic systems (Fialho et al., 2018), the relative humidity in Viçosa is higher during the beginning of the dry season and decreases toward the beginning of the rainy season, when it starts rising again (Fialho et al., 2018; Figure 3). Because of this pattern, some months with high daily relative humidity (e.g., May and June) have less snake activity than some months with lower daily relative humidity (e.g., October and November). This may reflect the greater influence of temperature on snake phenology, or due to unexplored variables, such as prey availability and reproductive cycle. Although daily relative humidity varies along the year in Viçosa, it is usually not below 70% (Figure 3), making this region humid year‐long.

Most specimens received at the MZUFV over a decade were captured from October to April (Figure 4; Figure S1), coinciding with the rainy season in the interior Atlantic Forest, which is normally warm and wet (Minuzzi et al., 2007). However, we did not find an effect of daily rainfall on the activity pattern of snakes. In southern Brazil, where rainfall is less seasonal, this variable is also not related to snake activity (Di‐Bernardo et al., 2007; Rocha et al., 2014). On the contrary, in southeastern (mainly coastal) Atlantic Forest, where a rainy season is well‐marked, rainfall has been reported to influence snake activity (Barbo et al., 2011; Hartmann et al., 2009a, 2009b; Marques et al., 2001). However, statistical testing of the influence of daily rainfall on the activity of snake communities has been rare in these studies (Hartmann et al., 2009b), and none used circular analyses, despite the periodic nature of the data being analyzed (Pewsey et al., 2013). Furthermore, rainfall is subject to more abrupt fluctuations in the interior Atlantic Forest throughout the calendar year, and snake activity there is mostly driven by environmental factors prone to milder fluctuations, such as temperature and relative humidity (Figure 3). Water availability directly or indirectly influences the incidence of prey (Siqueira et al., 2021; Zanella & Cechin, 2009) and helps snakes to cool themselves (Kearney et al., 2013). Most snake species from Viçosa feed on anurans (Costa et al., 2010), whose phenology overlaps with that of their predators (Pires & Feio, 2017). In summary, prey availability and other factors such as reproductive activity during warmer and wetter months (Marques et al., 2001; Pizzatto et al., 2007) may increase the seasonal activity of snakes during the rainy season, but on a daily basis, rainfall does not influence their phenology in the interior Atlantic Forest.

Climate‐driven phenological shifts have been consistently reported globally for different taxa in the past years (Benard, 2015; Forrest, 2016; Iler et al., 2021; Peñuelas et al., 2004; Visser & Both, 2005). Although changes in temperature and precipitation have also been observed and predicted for southeastern Brazil (Coltri et al., 2019; Perazzoli et al., 2013), we did not observe shifts in snake activities in Viçosa and vicinities over the decade analyzed. Nonetheless, we show increasing snake activity with temperature and precipitation, and therefore, the raising of temperatures and droughts may result in species shifting their activity times in the future. For instance, projected scenarios (based on the SSP585 scenario for three generalized circulation models [CNRM‐CM6‐1, IPSL‐CM6A‐LR, MRI‐ESM2‐0]; Fick & Hijmans, 2017) for the year 2050 in Viçosa and vicinities indicate a potential rise of 1.75–2.72°C in average annual temperature and a reduction of 44.5–87.0 mm in annual precipitation for the area. Constant monitoring and updated analyses are warranted given its potential impact on public health and on species survival in a rapidly changing world.

Three of the most common species recorded here are highly venomous: the pitvipers *Bothrops jararaca* and *B. jararacussu*, and the coral snake *Micrurus frontalis*. Another highly venomous species, the rattlesnake (*Crotalus durissus*), is expanding its distribution in the region, benefited from deforestation and climate change (Guerra et al., 2022) and was first recorded in Viçosa in 2015. This finding shows increased risks of snakebites in the region (Figure S2), and warrants attention of decision‐makers given its public health implications (de Almeida et al., 2022; Melo Araújo et al., 2022). For instance, snakebites—a tropical neglected disease (Minghui et al., 2019)—have increased in the Brazilian state of Minas Gerais (where Viçosa is located) in recent years (de Almeida et al., 2022). It also deserves noting the records of two non‐venomous snakes introduced by the pet trade: the corn snake (*Pantherophis guttatus*) and the red‐tailed boa (*Boa constrictor*), widely offered illegally on the internet (Nehemy et al., 2022). Records of free‐ranging *P. guttatus* (native from North America) are increasing (Fonseca et al., 2019), with high probability of becoming an invasive species in the Atlantic Forest (Fonseca et al., 2017). *Boa constrictor*, despite being a native species in Brazil, is not considered native to Viçosa and neighboring cities (Costa et al., 2010; Moura et al., 2012), so it could represent a threat to some of the native fauna in the region through competition or predation interactions. Our results support the importance of citizen science programs for monitoring and studying the impacts of introduced species, one of the main threats to biodiversity worldwide (Pyšek et al., 2020).

We highlight that more than 75% of all snake species known to occur in Viçosa (Costa et al., 2010) were recorded by local collectors during a decade of snake receptions. This finding stresses the importance of both biodiversity institutions and citizen science programs to improve our knowledge of biodiversity. Snakes are usually seen as dangerous and deceitful by many people, and end up being killed due to misinformation and fear (Lima et al., 2018; Lima‐Santos et al., 2020). Environmental education programs and science outreach initiatives can mitigate the aggressive behavior of humans toward snakes (Moura et al., 2010). Indeed, such initiatives have long been performed by the MZUFV staff, focusing on demystifying snakes and raising awareness of local people about the importance of these animals to ecosystem services, besides disclosing and stressing the role and importance of museums, natural history collections, and scientific research (Oliveira et al., 2009). These initiatives contribute to a greater engagement of citizen scientists and help, for instance, to document snake diversity through a low‐cost and educative approach, highlighting the importance of local collectors for biodiversity monitoring. Besides contributing to fill data gaps on the natural history and geographic distribution of reptiles, citizen scientists can also help to boost the discovery of undescribed species (Guedes et al., 2020; Moura et al., 2018), which is essential given the current biodiversity crisis (e.g., Ceballos et al., 2020).

Overall, we have shown that snake activity patterns in the interior Atlantic Forest are seasonal and mainly influenced by temperature and relative humidity. The peak of snake activity coincides with periods of high frequency of snakebites (de Almeida et al., 2022; see also Figure S2), which raises concerns for public health. This period also overlaps with school break and coffee harvest season in rural areas, one of the main economic activities in the region, which further aggravates the risk of snakebites. To raise awareness among local people about the risks of snakebite incidents and hospitalizations, it is important to implement environmental education programs (Moura et al., 2010) and promote collaboration between researchers and local decision‐makers from public health and environmental departments (Melo Araújo et al., 2022). This can help to prevent snake‐human conflicts, which usually results in the unnecessary killing of snakes.

## AUTHOR CONTRIBUTIONS

**Letízia M. G. Jesus:** Data curation (equal); writing – original draft (equal); writing – review and editing (equal). **Jhonny J. M. Guedes:** Data curation (equal); formal analysis (equal); methodology (equal); visualization (equal); writing – review and editing (equal). **Mario R. Moura:** Funding acquisition (equal); visualization (equal); writing – review and editing (equal). **Renato N. Feio:** Supervision (equal); writing – review and editing (equal). **Henrique C. Costa:** Conceptualization (equal); data curation (equal); supervision (equal); writing – review and editing (equal).

## CONFLICT OF INTEREST STATEMENT

The authors declare that they have no known competing financial interests or personal relationships that could have appeared to influence the work reported in this paper.

## Supporting information


Appendix S1.

## Data Availability

Raw data and R‐code needed to replicate the findings of this study are available at Zenodo Digital Repository: https://doi.org/10.5281/zenodo.8104430 (Jesus et al., 2023).
