# Supplementary material for: Environmental drivers of tropical forest snake phenology: Insights from citizen science
Source: Ecol Evol. 2023 Jul 23;13(7):e10305. doi: 10.1002/ece3.10305 (PMC10363785; doi:10.1002/ece3.10305)
Supplement: Supplementary file 1 — Appendix S1. [file ECE3-13-e10305-s001.docx]

**SUPPLEMENTARY MATERIAL**

**Environmental drivers of tropical forest snake phenology: insights from citizen science**

Letízia M. G. Jesus^1,2,†*^, Jhonny J. M. Guedes^3,†^,
Mario R. Moura^4,5^, Renato N. Feio¹ and Henrique C. Costa^6^

* Corresponding author. E-mail: letiziamgomes@gmail.com.

† These authors contributed equally to this work.

**This PDF file includes:**

Supplementary Tables

Supplementary Figures

SUPPLEMENTARY FIGURES

**Figure S1. Snake reception dates based on local collectors’ efforts in the interior Atlantic Forest of southeastern Brazil.** Circular plots show the distribution of all snakes (left) as well as venomous (center) and mildly/non-venomous snakes (right) received at the MZUFV collection throughout the calendar year over a decade. The arrows show the median (grey) and mean (black) direction, and the mean resultant length (a concentration metric), where shorter arrows indicate more dispersed snake reception dates. Data is from specimens collected in Viçosa and neighbor municipalities combined.

**Figure S2. Snakebite incidence and its relationship with snake activity in the study region.** a) Bar plots show the total number of snakebite reports in Viçosa and its seven neighbour municipalities between 2009 and 2018 according to SINAN – Sistema de Informação de Agravos de Notificação (information available at: http://portalsinan.saude.gov.br/sinan). b) The relationship between snakebites and snake activity (based on the citizen science data used in this study) across month-year combinations in the study region. The inset values show Pearson’s correlation coefficient.

SUPPLEMENTARY TABLES

**Table S1.** Variation Inflation Factor (VIF) among continuous variables included in the circular mixed-effects models.

| **Variables** | **Viçosa only** | **Viçosa + neighbours** |
| --- | --- | --- |
| Precip. | 1.251451 | 1.255094 |
| Avg. temp | 1.225334 | 1.214321 |
| Rel. humid | 1.466347 | 1.467751 |

**Table S2.** Model fit statistics for the intercept-only and the full models applied to the specimen data from Viçosa only and to Viçosa and neighbouring cities combined.

|  | **Viçosa-only** | | **Viçosa + neighbours** | |
| --- | --- | --- | --- | --- |
| **Criterion** | **Intercept-only** | **Full model** | **Intercept-only** | **Full model** |
| DIC | 1355.5 | 1098.5 | 1483.0 | 1198.6 |
| DICalt | 1380.4 | 1161.9 | 1501.8 | 1264.4 |
| WAIC1 | 1359.0 | 1114.0 | 1486.6 | 1220.5 |
| WAIC2 | 1359.2 | 1114.7 | 1486.8 | 1221.2 |

**Table S3.** Results from the sensitivity analysis, where precipitation was used as the summed values over the prior week to snake capture dates. Posterior modes in radians (and degrees) and 95% highest posterior density (HPD) interval, with lower (LB) and upper bounds (UB) for the circular regression SAM coefficients for the continuous variables of the snake reception data. Bold values indicate that an HPD interval does not contain 0. Predictors were z-transformed to make them comparable. Data based on specimens collected on Viçosa only.

| **Predictors** | **mode** | **sd** | **LB** | **UB** |
| --- | --- | --- | --- | --- |
| Precip.z | -1.87 (-107) | 636.9 | -42.00 | 37.80 |
| AvgTemp.z | 1.57 (90.4) | 4.9 | **0.92** | **4.60** |
| RelHumid.z | 1.01 (58) | 0.4 | **0.57** | **1.94** |
